# Networks of cortical activity in infants with epilepsy

**DOI:** 10.1093/braincomms/fcac295

**Published:** 2022-11-05

**Authors:** Sami Auno, Henna Jonsson, Tarja Linnankivi, Anton Tokariev, Sampsa Vanhatalo

**Affiliations:** BABA Center, Department of Clinical Neurophysiology, New Children’s Hospital, Helsinki University Hospital, Helsinki, 00029 HUS, Finland; Neuroscience Center, Helsinki Institute of Life Science, University of Helsinki, 00014 Helsinki, Finland; Epilepsia Helsinki, University of Helsinki and Helsinki University Hospital, Helsinki, 00029 HUS, Finland; Department of Physiology, University of Helsinki, 00014 Helsinki, Finland; Epilepsia Helsinki, University of Helsinki and Helsinki University Hospital, Helsinki, 00029 HUS, Finland; Department of Pediatric Neurology and Pediatric Research Center, New Children’s Hospital, Helsinki University Hospital and University of Helsinki, Helsinki, 00029 HUS, Finland; Epilepsia Helsinki, University of Helsinki and Helsinki University Hospital, Helsinki, 00029 HUS, Finland; Department of Pediatric Neurology and Pediatric Research Center, New Children’s Hospital, Helsinki University Hospital and University of Helsinki, Helsinki, 00029 HUS, Finland; BABA Center, Department of Clinical Neurophysiology, New Children’s Hospital, Helsinki University Hospital, Helsinki, 00029 HUS, Finland; Neuroscience Center, Helsinki Institute of Life Science, University of Helsinki, 00014 Helsinki, Finland; Department of Physiology, University of Helsinki, 00014 Helsinki, Finland; BABA Center, Department of Clinical Neurophysiology, New Children’s Hospital, Helsinki University Hospital, Helsinki, 00029 HUS, Finland; Neuroscience Center, Helsinki Institute of Life Science, University of Helsinki, 00014 Helsinki, Finland; Department of Physiology, University of Helsinki, 00014 Helsinki, Finland

**Keywords:** functional connectivity, EEG, epilepsy, infants

## Abstract

Epilepsy in infancy links to a significant risk of neurodevelopmental delay, calling for a better understanding of its underlying mechanisms. Here, we studied cortical activity networks in infants with early-onset epilepsy to identify network properties that could pre-empt infants’ neurodevelopmental course. We studied high-density (64 channel) electroencephalogram during non-rapid eye movement (N2) sleep in *n* = 49 infants at 1 year of age after being diagnosed with epilepsy during their first year of life. We computed frequency-specific networks in the cortical source space for two intrinsic brain modes: amplitude–amplitude and phase–phase correlations. Cortical activity networks of all frequency bands and connectivity modes were compared between the syndrome groups as well as between the three categories of neurocognitive development. The group differences were studied at three spatial levels: global, regional, and individual connections. Cortical mechanisms related to infant epilepsy were further compared with physiological networks using an automatic spindle detection algorithm. Our results show that global connectivity does not significantly differ between epilepsy syndromes; however, it co-varies with neurocognitive development. The largest network differences were observed at the lowest (<1 Hz) and mid-range (10–15 Hz) frequency bands. An algorithmic removal of sleep spindles from the data partially reduced the mid-range frequency network’s strength. The centrocentral and frontocentral networks at the spindle frequencies were found to be strongest in infants with a persistent age-typical neurocognitive performance, while their low-frequency (< 1 Hz) networks were weaker for both amplitude-amplitude [*P* = 0.008, effect size = 0.61] and phase–phase correlations (*P* = 0.02, effect size = 0.54) at low (< 1 Hz). However, subjects with persistent mild neurocognitive delay from 1 to 2 years of age had higher amplitude–amplitude (*P* = 0.02, effect size = 0.73) and phase–phase (*P* = 0.06, effect size = 0.59) at low frequencies than those that deteriorated from mild to severely delayed from 1 to 2 years of age. Our findings suggest that cortical activity networks reflect the underlying clinical course of infants’ epilepsy, and measures of spectrally and spatially resolved networks might become useful in better understanding infantile epilepsy as a network disease.

## Teaser

Auno *et al.* report that cortical activity networks examined from the scalp electroencephalogram provide a sensitive measure of brain function. Infants with epilepsy exhibited specific network changes that link to the severity of both current and upcoming neurological outcomes. They may be used for guiding early interventions in infants with epilepsy.

## Graphical Abstract

**Graphical abstract:**
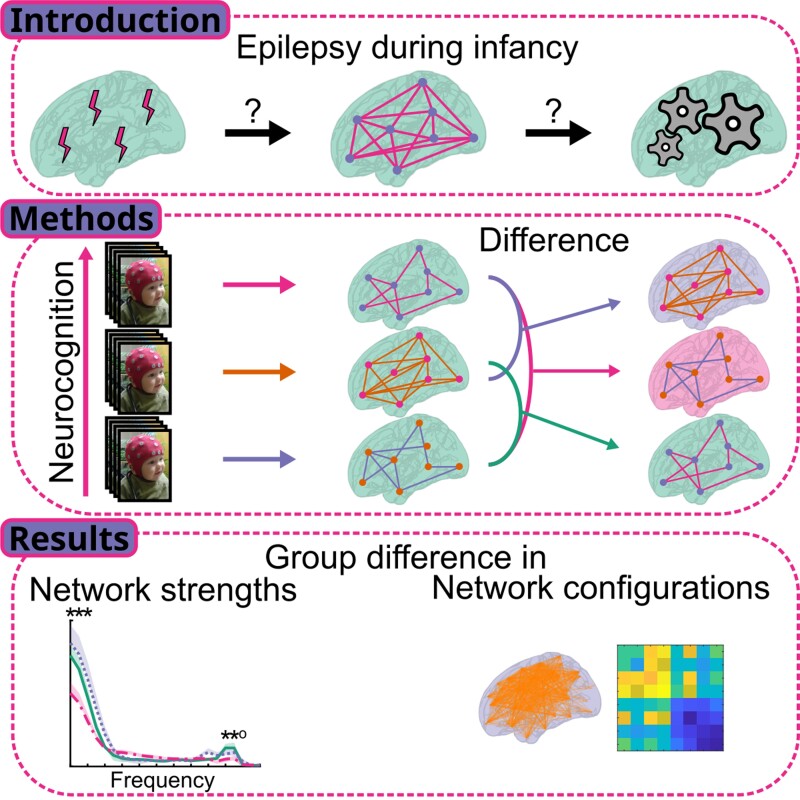

## Introduction

Epilepsy in infancy is a common neurological condition with an incidence of 1.2 per 1000 live births during the first year of life.^1^ Epilepsy presents a high risk for the neurodevelopmental delay;^2^ however, the developmental consequences are highly dependent on a multifactorial interplay between the aetiology of the specific epilepsy syndrome, age of seizure onset, and the use of and response to antiepileptic medication. The precise mechanisms and relationships between epilepsy, aetiology, seizure outcome, and neurocognitive delays remain poorly understood, and they present an everyday diagnostic challenge for clinicians.

Recent works on network neuroscience have characterized epilepsy as a network disease.^3–5^ The ubiquitous, large-scale correlations in spontaneous brain activity are known to underly many higher brain functions,^6^ but they also participate in the pathophysiology of many neurological disease states, such as epilepsy. Prior research has shown that epileptogenesis may involve the growth of aberrant brain circuitries, resulting in large-scale network instability and characteristic shifts between ictal and interictal states.^7,8^ Notably, such changes are also considered to contribute to poor neurocognitive outcomes in many neurological conditions.^9–11^

Characterizing infant epilepsy as a network disease^3^ could offer some important benefits: first, it would allow employing a large set of network neuroscience tools and frameworks developed in the adult and experimental literature.^12–15^ Second, it would build on the notion of global or generalized brain dysfunction, which is perhaps closer to reality in the case of infant epilepsies than the commonly employed localization-related structural or electrophysiological measures.^16,17^ Third, analysing brain networks is commonly done from resting-state activity, which is readily available in infants during sleep,^18–20^ making the approach clinically achievable.

Here, we set out to study how networks of cortical activity reflect the underlying, newly diagnosed epilepsy in infants at 1 year of age. To this end, we developed a pipeline with a realistic source model to analyse phase- and amplitude-based correlations in cortical activity across a wide range of oscillatory frequencies. We compared the cortical networks between epilepsy syndromes and groups of neurocognitive developments.

## Materials and methods

**Figure 1:**
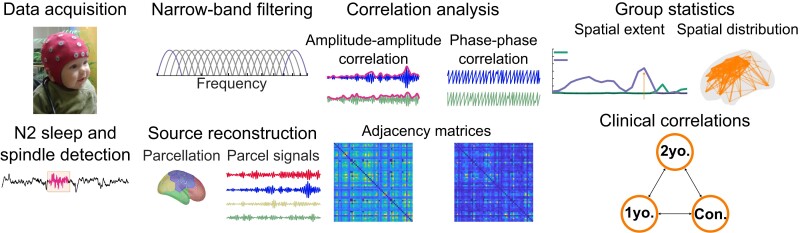
**An overview of the study design and analysis methods.** Sixty-four-channel high-density EEG was measured from 49 1-year-old infants. Epochs with N2 sleep were identified visually, and the sleep spindles were automatically detected. The data were then band-pass filtered to 25 frequency bands and inverse modelled to cortical source signals, subsequently collapsed to 58 cortical parcel signals. Network interactions were estimated from AAC and PPC between each parcel. The connectivity strength is visualized as adjacency matrices. The group-level comparisons were made for each frequency band for three neurocognitive outcomes at 1 to 2 years of age and five groups of epilepsy syndromes.

An overview of the methods are presented in Fig. 1.

### Study cohort

We analysed daytime sleep electroencephalograms (EEGs) from 49 1-year-old infants (median age 12.4 months with an interquartile range between 12.3 and 12.8 months, Table 1) in the New Children’s Hospital, Helsinki University Hospital, Finland. We prospectively recruited infants from the Helsinki University Hospital Specific Catchment Area (2.2 million inhabitants in 2020) with an epilepsy diagnosis between February 2017 and May 2019 and seizure onset before 12 months of age. In total, we recruited 63 infants. The inclusion criteria for the present analysis were as follows: (i) high-density EEG (64 channels) at 1 year of age, (ii) over 3 minutes of N2-state sleep in the EEG, and (iii) neurological assessment at 1 to 2 years of age. Of the 63 subjects, 49 passed these criteria. Of the 14 not included, 11 had a low-density EEG at 1 year of age, one did not have enough N2-state sleep, one had hypsarrhythmia during sleep measurements, and one passed away before 2 years of age. This study was approved by the Ethics Committee at the Helsinki University Hospital, and informed written consent was received from the guardians before the study’s inclusion.

**Table 1:** Clinical information of the subjects. The number of subjects, the gender ratio, and the mean age at 1 year EEG in the different subgroups

Subgroup	Number of subjects	Gender (% female)	Age at 1 year EEG (months ± SD)
All subjects	49	51.0	12.5 ± 0.8
Epilepsy syndrome			
Self-limited	12	41.7	12.4 ± 0.8
West syndrome	27	48.1	12.5 ± 0.6
Unclassified focal	8	62.5	12.3 ± 0.8
Other	2	100.0	12.9 ± 0.8
Neurocognitive development 1 year			
Typical	20	45.0	12.4 ± 0.9
Mild	10	80.0	12.3 ± 0.8
Severe	19	42.1	12.7 ± 0.5
Neurocognitive development 2 years			
Typical	14	50.0	12.4 ± 0.9
Mild	10	50.0	12.4 ± 0.8
Severe	25	52.0	12.6 ± 0.5

### Clinical grouping

We grouped the infants in two ways: first, they were classified by their epilepsy syndrome and grouped into four categories: self-limited epilepsy syndrome (infantile, neonatal, and neonatal-infantile); west syndrome (infantile spasms syndrome); unclassified focal epilepsy; and other. Second, we assessed the global neurocognitive delay of the infants and grouped them into three groups: age-typical development (Typical), mildly delayed (Mild), and severely delayed (Severe). The epilepsies were defined and classified according to the International League Against Epilepsy (ILAE) position papers^21,22^ on the definition and classification of the epilepsies based on all clinical data, EEG investigations, and aetiological evaluations performed. Epilepsy syndromes were defined using the ILAE’s proposed 2021 classification guideline: syndromes in neonates and infants.^23^ Data on neurocognitive developments were based on clinical evaluation by an experienced paediatric neurologist (H.J.), the Griffiths Scales of Child Development,^24^ and the Hammersmith Infant Neurological Examination at 12 and 24 months of age. In addition, at 24 months, a neuropsychological evaluation was performed with the Bayley Scales of Infant and Toddler Development (BSID-III).^25^

We defined development as typical if the test scores were better than or equal to −1 standard deviations of the test normative mean (BSID-III) or the developmental quotient was above or equal to 80 (Griffiths). We defined development as mildly delayed if the test score was below −1 but above or equal to −2 standard deviations of the norm in all domains or lower than −2 standard deviations in one domain, provided that scores in the other domains were within age-appropriate development (BSID-III) or the developmental quotient was between 70 and 79 (Griffiths). We defined severe delay as test scores below −2 standard deviations of the norm (BSID-III) or the developmental quotient below 70 (Griffiths) or clinically observed vast global delay in all areas, so that formal assessment by Bayley III was not applicable.

### Electroencephalogram recordings and epoch selection

The EEG signals were recorded with an eego EEG amplifier (ANT-Neuro, Germany) at a sampling rate of 500 Hz using 64 channel EEG caps (sintered Ag/AgCl electrodes; Waveguard, ANT-Neuro, Germany) positioned according to the International 10–5 standard. AFz was used as an original reference in all recordings. We converted the data to European Data Format for visual review and epoch selection in the clinical NicOne Reader software (Natus, USA) and further signal processing and analysis using our in-house scripts based on MATLAB (MathWorks, Natick, MA, USA).

We reviewed the EEG data visually to verify artifact-free epochs and channels, as well as to select the epochs with N2 sleep using the following criteria: N2-epoch was chosen from the beginning of the first unequivocal sleep spindle and continued until the occurrence of another sleep state (N1, N3, or arousal) as defined per conventional EEG criteria.^26^ The median length of N2 sleep analysed was 10.0 ± 5.2 minutes (median ± standard deviation minutes) per patient.

### Data preprocessing

#### Electroencephalogram preprocessing

A detailed flowchart of the preprocessing pipeline is presented in Supplementary Figs. 1 and 2. Here, we briefly summarize this pipeline. The EEG data were band-pass filtered to a 0.15–48 Hz frequency band and downsampled to a sampling rate of 250 Hz. We replaced channels with no contacts with spherical spline interpolation.^27^ The median number of interpolated bad channels per subject was 3. After interpolating bad channels, we utilized an automated spindle detection algorithm to flag spindle segments (see below). The EEG data were then re-referenced to a common average reference montage and narrow band-pass filtered into 25 linearly spaced frequency bands with central frequencies (Fc) ranging from 0.5 to 39.7 Hz. We set the first Fc to 0.5 Hz, and all consecutive central frequencies were computed as 1.2 times the previous Fc. All frequency filtering was done by applying a combined low-pass/high-pass Kaiser window finite impulse response filter as implemented in the Brainstorm^28^ MATLAB toolbox with an attenuation of 40 dB, a stopband of [0.5*Fc*, 1.5*Fc*], and a cut-off of [0.85*Fc*, 1.15*Fc*].

#### Spindle detection

We used a custom adaptation of an automated spindle detection algorithm widely used in previous studies.^29–32^ In order to limit false positive detection, we calculated the sum of spindle signal power over all channels and flagged segments as spindles only if the spindle power during that segment exceeded a certain threshold. The threshold was based on the mean peak amplitude of the spindle power envelope. See the Supplementary material online and Supplementary Fig. 3 for a more detailed description of the spindle detection pipeline.

#### Source reconstruction

We used, a publicly available common head model based on 90 MRI scans of 1 year old infants^33^ in conjunction with openMEEG.^34,35^ Based on our previous studies,^36,37^ we chose the tissue conductivity parameters of 1.79, 0.1, and 0.43 S/m for intracranial volume, the skull, and the scalp, respectively. Source space reconstruction was performed with the Dynamical Statistical Parametric Mapping.^38^ We used an identity matrix for the noise covariance matrix that considers the same noise level in all recording electrodes.

The model estimated 8562 cortical dipole source signals. These were then collapsed into 58 parcel signals, representing the mean cortical activity within each parcel.^10^ The detailed description of this method was summarized by Korhonen *et al*.^39^ and Tokariev *et al*.^10^

#### Estimating cortical network connectivity

There are multiple alternative approaches for studying the activity networks in humans.^40–43^ We estimate activity networks with amplitude and phase interactions between source activity in cortical regions as measured with EEG and inversely modelled to the cortex. The interareal amplitude and phase dynamics between cortical regions reflect different facets of their neuronal interactions. The amplitude–amplitude correlations (AACs) are thought to arise from the comodulation of general neuronal activity and gross cortical excitability over periods of seconds,^6,44^ whereas the phase–phase correlations (PPCs) reflect the neuronal communications at millisecond scale temporal precision.^10,45,46^

A network consists of nodes and edges. Here, each parcel is considered a node, and the functional coupling between two parcel signals is considered an edge. In order to estimate the AAC between cortical regions, we employed the orthogonal correlation coefficient (oCC).^12^ Orthogonalization minimizes spurious correlations that are caused by volume conduction.^12^ The AACs were estimated by taking the Pearson correlation coefficient between the amplitude envelopes of orthogonalized signals. We used non-overlapping 1-s windows to orthogonalize the Hilbert transformed parcel signals relative to each other. We performed this procedure in both directions for each parcel pair: signal *x* was orthogonalized relative to signal *y*, and signal *y* was orthogonalized relative to *x* These two correlation coefficients were then averaged for each parcel pair (*x* and *y*). The oCC values are scaled between −1 and 1.

In addition to amplitude-amplitude coupling, we also estimated the PPC between cortical regions. We applied a debiased estimator of the squared weighted phase lag index (dwPLI).^13^ The dwPLI is insensitive to volume conduction as it ignores phase lags close to zero-phase.^47,48^ The dwPLI values are scaled between 0 and 1.

#### Network correction

The correlation coefficients between all possible parcel pairs resulted in 1653 pairwise interactions. We corrected the resulting correlation matrices by excluding those connections that could not be accurately estimated using 64 channel EEG recordings. In order to define such connections, we simulated artificial parcel activity so that each parcel pair was synchronized at a time. We then computed a synthetic EEG by using the forward solution on the simulated parcel signals and then reconstructed the parcel signals from the synthetic EEG with the inverse solution. These reconstructed parcel signals were correlated with the original artificial parcel activity. This process was repeated 500 times for each parcel pair. We then compared the pairwise interactions of reconstructed parcels initially in synchrony to a set of surrogate values from all non-synchronous parcels from all iterations. We then rejected those interactions below the 99th percentile of the surrogates. For a more detailed description of this process, see elsewhere.^10^ We excluded 24 (1.45%) of the interactions from all adjacency matrices.

#### Statistical analysis

We examined the mean global connectivity at different frequency bands. The mean global connectivity was calculated by taking the mean of all AAC or PPC edges of all subjects within a given group at a given frequency band. We compared the mean global connectivity between the groups and determined the statistical significance with a Kruskal–Wallis one-way ANOVA. In addition to comparing the mean global connectivity strength between the groups, we also compared the edge-level differences between the groups at each frequency band. We performed this comparison by first applying the Wilcoxon rank-sum test (one-tailed test with *α* = 0.05) edge-by-edge in both directions (e.g. Typical > Mild and Mild > Typical). This procedure was performed for both AAC and PPC on each frequency band and group pair (Typical-Mild, Typical-Severe, Mild-Severe). We corrected the resulting *P*-value matrices for false discovery rates using the Benjamini–Hochberg method with *α* = 0.05. The extent of the higher activity network was expressed as network density (*K*),^49^ which gives the portion of significantly stronger edges out of the total number of edges in the network.

## Results

### Global mean connectivity

**Figure 2:**
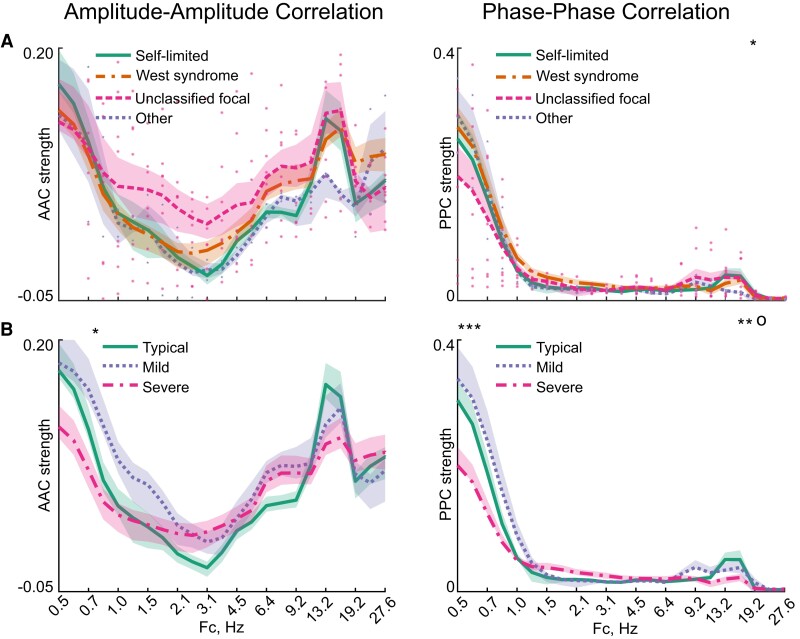
**Connectivity strengths as a function of oscillatory frequency.** Global mean AAC and PPC strengths in the four epilepsy syndrome groups (**A**) and the three neurocognitive development groups (**B**) as a function of the Fc band. For groups with under 10 subjects individual data points are shown. Dots indicate subjects in the unclassified focal group (eight subjects) and the small stars indicate subjects of the other group (two subjects). The neurocognitive development groups were determined at the age of 2 years. The asterisk indicates the Fc at which the difference between the groups is significant with *P* < 0.05, whereas o indicates *P* < 0.1. *P*-values were determined with Kruskal–Wallis one-way ANOVA.

The mean global connectivity strengths in AAC and PPC networks were strongly related to oscillatory frequency (Fig. 2). Both connectivity modes exhibited strong connectivity at the lowest frequencies (< 1.5 Hz), particularly in the PPC. The global AAC levels exhibited a U-shaped relationship to frequencies, with a nadir at about 3 Hz. Both PPC and AAC networks showed an apparent peak at beta frequencies (9–19 Hz).

Global frequency-wise connectivity strengths were comparable between different epilepsy syndromes (Fig. 2A). The only statistically significant group difference was found at the PPC networks’ single beta frequency bin (Fc = 19.2 Hz, *P* = 0.03, false discovery rate corrected for 1653 pairwise interactions).

Global frequency-wise connectivity strengths were different between groups with different levels of neurocognitive development (Fig. 2B). The most robust group difference was seen between the subjects with a severe delay and other groups. Notably, the group differences were observed at the same frequency ranges for both AAC and PPC networks, with the most evident differences at low delta (<1 Hz) and beta (13–19 Hz) frequencies.

These findings jointly suggest a link between neurocognitive delay and the global strength of both AAC and PPC networks. However, the group-wise differences appeared to be less robust between syndrome groups than between groups with different neurocognitive developments, as we found no statistically significant differences at any frequency band in AAC between the syndrome groups, and only one statistically significant difference at frequency band Fc = 19.2 Hz in PPC.

### Effects of spindles on the global networks

The global frequency-wise analyses peaked at around Fc = 13 Hz in all groups, and this frequency range also exhibited significant differences between our clinical groups. This frequency is intriguingly close to the characteristic frequency range of sleep spindles, the hallmark of N2 sleep. Hence, it is easy to envisage that our findings may arise from the networks associated with sleep spindles. This idea was tested by comparing the full EEG data to signals where, after algorithmic event detection, sleep spindles had been removed.

The number of sleep spindles per unit time or the number of spindle groups per unit time did not differ between the neurocognitive groups (Supplementary Fig. 4A and B, *P* = 0.16, *ES* = 0.04 and *P* = 0.06, *ES* = 0.08, respectively). The spindle groups were slightly longer in the Typical group (1.4 s ± 0.7 s) than in the other groups (1.2 s ± 0.7 s and 1.1 s ± 0.6 s in Mild and Severe groups, respectively, *P* = 0.02, *ES* = 0.14) and the number of sleep spindles within each spindle group was correspondingly greater in the Typical group (15.0 s ± 9.6 s) compared with the other groups (11.9 s ± 10.2 s and 9.4 s ± 7.4 s in Mild and Severe groups, respectively, *P* = 0.02, *ES* = 0.14) (Supplementary Fig. 4C and D).

Removing sleep spindles from the EEG data reduces the group differences in the corresponding PPC and AAC networks (Supplementary Fig. 5): on the neurocognitive-wise comparison, the Severe group exhibited weaker connectivity at the low-frequency bands; however, the differences at spindle frequencies had disappeared. Similarly, the group differences diminished at Fc = 13 Hz on the syndrome-wise comparison.

### Spatial group difference

**Figure 3:**
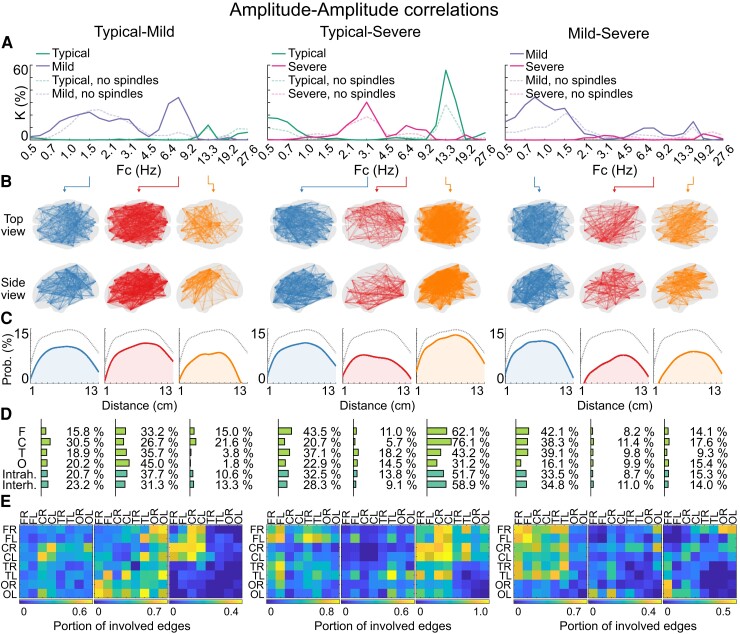
**Group comparison in AAC.** (**A**) Shows the portion of the edges (density, K) that are significantly stronger in the given group when compared with the other group specified. The dashed line shows the network density after removing of spindles from the data. The statistical analyses were performed with Wilcoxon rank-sum test for equal medians with *P*-value threshold of 0.05 and corrected for false discovery rate with Benjamini–Hochberg method. (**B**) Shows the significantly stronger edges in the given group comparison. In (**C**), the distributions of connection lengths are shown on a logarithmic scale. (**D**) Shows the proportion of network nodes per brain region; here, 100% involvement would signify that all possible edges from the given region are involved in the network. Likewise, in (**E**), the portion of edges is shown between two brain regions that are part of the difference network. 1.0 signifies that all edges are part of the difference network. C = central lobe; F = frontal lobe; Fc = central frequency; O = occipital lobe; T = temporal lobe; Interh. = interhemispheric; Intrah. = intrahemispheric.

**Figure 4:**
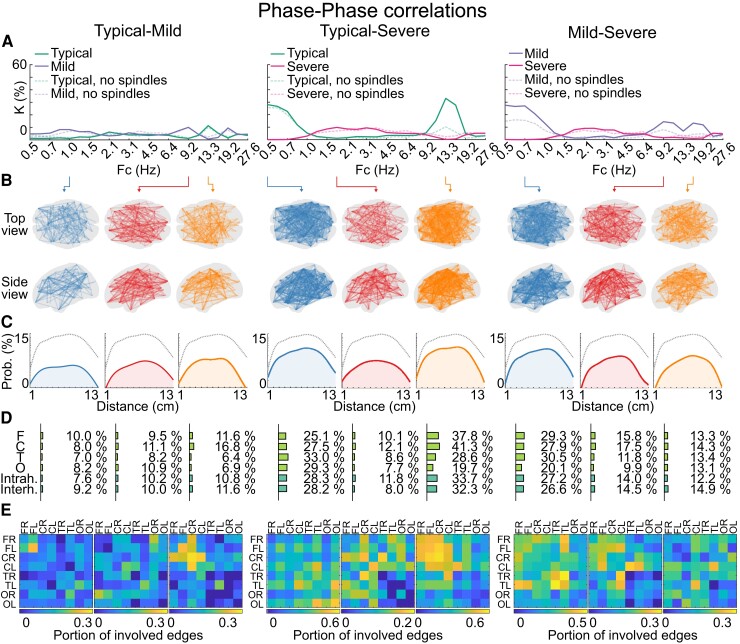
**Group comparison in PPC.** (**A**) Shows the portion of the edges (density, K) that are significantly stronger in the given group when compared with the other group specified. The dashed line shows the network density after removing of spindles from the data. The statistical analyses were performed with Wilcoxon rank-sum test for equal medians with *P*-value threshold of 0.05 and corrected for false discovery rate with Benjamini–Hochberg method. (**B**) Shows the significantly stronger edges in the given group comparison. In (**C**), the distributions of connection lengths are shown on a logarithmic scale. (**D**) Shows the proportion of network nodes per brain region; here, 100% involvement would signify that all possible edges from the given region are involved in the network. Likewise, in (**E**), the portion of edges is shown between two brain regions that are part of the difference network. 1.0 signifies that all edges are part of the difference network. C = central lobe; F = frontal lobe; Fc = central frequency; O = occipital lobe; T = temporal lobe; Interh. = interhemispheric; Intrah. = intrahemispheric.

To further investigate the spatial distributions of the group differences, we performed edge-by-edge comparisons between respective groups, and we calculated the extent of networks showing the significant difference (i.e. network density; Figs 3 and 4). For further analysis, we examined three different frequency bands from each clinical group comparison. The examined frequency bands were selected from the low (< 3 Hz), middle (3–13 Hz), and high (> 13 Hz) frequency bands. We chose those frequency bands that displayed the greatest network densities.

A comparison of the Typical group with the Mild and Severe groups showed stronger AAC and PPC connectivity at around 13 Hz in extensive networks that covered frontocentral regions (Figs 3B, 3C, 4B and 4C) and included 33% (PPC) to 56% (AAC) of all possible network edges. These also extended to the temporal region, involving less of the occipital nodes in the AAC (∼31%) and PPC (∼20%) networks (Figs 3D, 3E, 4D, and 4E). Strikingly, much of this network remained after detector-based removal of spindle epochs (Figs 3A and 4A, also Supplementary Figs 6 and 7), suggesting that it may involve oscillatory network mechanisms beyond the canonical sleep spindles.

A comparison of the Mild group with other groups showed stronger AAC and PPC connectivity at lower (< 3 Hz) and medium (5–9 Hz) frequencies. While the exact peak frequencies differed between comparisons, these networks were generally prominent in wide brain areas, covering frontal, central, and temporal regions. The removal of spindles affected these network comparisons (Figs 3A and 4A, also Supplementary Figs 6, 7, 8, and 9).

The Severe group displayed stronger networks at low and medium frequency bands, especially at around 3 Hz, with a frontotemporal emphasis in network distribution.

### Network correlations of developmental trajectories

We next grouped the infants according to their change in neurocognitive development from the first to the second year of life (for subject-wise details, see Supplementary Table 1). This resulted in five groups: Typical-Typical, Typical-Mild, Mild-Mild, Mild-Severe, and Severe-Severe. Only one infant progressed from Typical at 1 year of age to Severe at 2 years of age and was thus excluded from further analysis. See Supplementary Fig. 10 for group sizes at each age and the change in each group. Examining the change in neurocognitive development and the associated networks could yield a clinically meaningful prediction of the change in neurocognitive outcome from the early cortical network activity.

**Figure 5:**
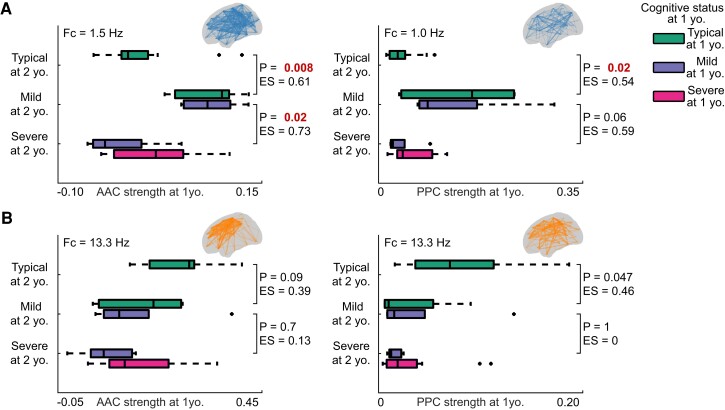
**Comparison of the connectivity strengths between groups by changes in neurocognitive development.** The subjects have been grouped into five groups based on the change in the neurocognitive performance from 1 to 2 years of age. The bars signify performance at one year of age while the neurocognitive development at 2 years of age is labelled on the y-axis; the x-axis depicts connectivity strengths with AAC on the left column and PPC on the right column. The connectivity strength is the mean over the shown edges (see the glass brain in the upper right corner) on the given Fc. Two frequency regions are presented: low frequencies on (**A**) and higher frequencies on (**B**). The statistics are shown for comparisons where groups have had the same neurocognitive development at 1 year of age but different at 2 years of age (Wilcoxon rank-sum with ES indicated. All *P*-values are corrected for false discovery rate with Benjamini–Hochberg method).

Infants with persisting age-typical neurocognitive performance displayed generally weaker AAC at lower frequencies (< 10 Hz) compared with those with a change from age-typical to mildly delayed performance from 1 to 2 years of age (Supplementary Fig. 1^1^). This group difference was largest at Fc = 1.5 Hz in AAC (*P* = 0.008, *ES* = 0.61 (Fig. 5)) and at Fc = 1.0 Hz in PPC (*P* = 0.02, *ES* = 0.54). The Fc = 1.5 Hz AAC network differentiated those subjects that were to develop mild cognitive impairment by 2 years of age well: taking the mean AAC network strength of 0.03 at Fc = 1.3 Hz as a threshold, this would have a sensitivity of 1 and specificity of 0.86 in differentiating those 1 year old subjects that were to develop mild neurocognitive delay.

On the other hand, subjects with a persisting Mild neurocognitive delay from 1 to 2 years of age (*N* = 5) displayed stronger AAC and PPC strengths across a wide frequency range compared with those that deteriorated from Mild to Severe delay by 2 years of age (*N* = 5) (Supplementary Figs. 11 and 12). Again, the difference was largest at Fc = 1.5 Hz in the AAC networks (*P* = 0.02, *ES* = 0.73) (Fig. 5), though only trending in the PPC networks (*P* = 0.06, *ES* = 0.59). Infants with persistent Severe neurocognitive delay (*N* = 19) were indistinguishable from those infants with Mild delay at 1 year of age but Severe delay at 2 years of age (*N* = 5) at all studied frequency bands.

## Discussion

Our study shows that spontaneous cortical activity in infants with epilepsy exhibits large-scale AAC and PPC networks with spectrally and spatially specific constellations that may distinguish between neurodevelopmental trajectories. Our results are generally consistent with the prior studies on older children and adults reporting large-scale cortical networks that correlate with the level of neurocognitive delay in patients with epilepsy.^50–55^ Here, we extend prior knowledge by showing comparable network effects and clinical correlations in infants, suggesting a high ontogenetic resilience of the associated network mechanisms. Furthermore, we showed that the group differences in the networks might be partly assigned to sleep spindles, which corroborates other recent works on the importance of sleep spindles in predicting the evolution of paediatric epilepsies.^56,57^ Taken together, the findings suggest that sleep spindles may provide an essential, endogenous framework for the cortico-cortical activity network during sleep.

Epilepsy is commonly thought to be a stereotypical network disorder.^5^ Epileptic seizures and interictal discharges are generated and spread in networks that range spatially from very local to global networks encompassing both hemispheres.^55,58^ Arguably the most evident consequence of the network nature of epilepsy is the spread of ictal activity during a seizure. The networks involved in the ictal spread are thought to explain the semiology of the seizure,^59,60^ which is utilized in localization of the symptomatogenic zone(s) and thus aids in the presurgical evaluation of epilepsy surgery.^59,60^ In addition to ictal networks, a growing body of evidence shows that the resting-state, non-spiking network connectivity is also altered in epilepsy.^55,61–63^ These altered resting-state networks could be involved in generating and propagating interictal discharges and seizures, or they could reflect cognitive changes during the clinical course of epilepsy. Recent studies on adult patients with epilepsy have reported a more extensive set of epilepsy-related network alterations: For instance, non-spiking EEG and MEG data in brain areas linked to epileptic foci are reported to exhibit increased connectivity strengths compared with corresponding regions in healthy controls.^55,61^ Additionally, patients with either focal or primarily generalized epilepsy are found to have altered connectivity in various networks associated with strong inter- or intraregional connectivity in healthy controls, such as the default mode network,^55,62^ the somatomotor network,^62,63^ the ventral attention network,^62^ and networks involving the anterior cingulate cortex.^55,61,62^ Our present findings in infants show a range of group difference networks, which supports the overarching conclusion from the prior literature that epilepsy affects diverse networks with as yet only partially explained clinical correlations.

Although there is no straightforward universal convergence between the reported epilepsy-related network patterns, the network effects may still relate to individual level clinical factors such as disease duration or cognitive dysfunction.^52,55^ For instance, Coito *et al.*^55^ showed reduced connectivity from the anterior cingulate cortex in patients with temporal lobe epilepsy suffering from learning difficulties compared with patients without learning difficulties. These observations are broadly concordant with our findings that frontocentral connectivity at alpha and low beta bands is stronger in infants with age-typical neurocognitive development levels compared with infants with a neurocognitive delay. Similar findings have also been reported in fMRI and DTI studies.^64–70^ Although these frontocentral differences were the most apparent at the sleep spindle frequencies in our data set, removing sleep spindles was not enough to remove these differences. Together with the previous reports, these findings indicate that both functional and structural alteration of these networks occurs in epilepsy and that they are linked to the cognitive outcomes in patients with epilepsy.

The best known developmental effects in the sleep EEG activity during the infantile period include a gradual resolution of the occipitofrontal amplitude gradient at the slow frequencies, the gradual shortening of sleep spindles, as well as an increase in their interhemispheric cooccurrence.^71^ Our present results extend the existing literature on the amplitudes and graphoelements, and we suggest that the EEG oscillations exhibit robust correlation structures in both the amplitude and phase domains. Moreover, these correlation structures are frequency-dependent, and they show clinical group differences. Prior studies have shown that interhemispheric coherence increases and intrahemispheric coherence decreases during childhood development.^72^ The coherence measures used in the prior studies are by default a mixture of amplitude and phase effects,^73^ which were studied separately in our work. The present group-wise comparisons between syndromes and neurocognitive developments showed no significant difference between the intra- and interhemispheric networks in either the AAC or the PPC networks. However, regional comparisons indicated that especially the centrocentral and frontocentral regions are typically involved in the group difference networks. These findings suggest that the specificity of the pathophysiological effects of infantile epilepsy is at the level of brain regions rather than hemispheres.

Our present study includes multiple simplifications that do not qualitatively influence the overall conclusions, but they may limit the generalizability of our findings. First, we included infants presenting with an epilepsy diagnosis before 1 year of age. The lack of healthy controls precludes assessment of the effects of epilepsy in general on the brain’s networks. However, epilepsy is already well established to be essentially a network disorder,^55,61–70^ and in clinical reality, the diagnosis of epilepsy is established by the time of the EEG recordings. Here, we aimed to search for additional information value in this clinical context, and thus examined the network differences within and between epilepsy syndromes and the clinical course of neurocognitive development, which are essential domains of interest in the clinical treatment of infants with epilepsy.

Second, our sample size was 49 infants, which is relatively low for statistical inferences, though it reflects a systematic population-based collection available in an active epilepsy unit that covers a population of about 2 million. The cohort size necessitated some compromise in group aggregates, which may have led to underestimating the actual group differences. This was most evident in the syndrome-wise grouping, as these group aggregates ended up representing rather broad clinical phenotypes.

Third, our preprocessing pipeline includes some compromise in the exact source localization, which results from using a common head model, visual placement of electrodes (instead of electrode digitization), and the use of equally parcelled, non-anatomical cortical source space. While there are clear practical, theoretical, and logistic reasons for such choices, it is also possible that some altered individual brain anatomy with network effects is overlooked in our approach. Obtaining individual MRIs or electrode digitizations is not ethically or practically feasible, while our pipeline was aimed at offering solutions that may be scaled to any clinical unit as needed. Although limited in anatomical accuracy, random parcellation allows the most balanced representation of the global network function recorded with scalp EEG.^10^

Our study shows that spontaneous, resting-state cortical activity as measured with high-density EEG exhibits large-scale amplitude and phase correlations that may spectrally and spatially differentiate the neurocognitive trajectories of infants with epilepsy. A prospective classification of infants with newly diagnosed epilepsy as high or low risk with respect to future neurocognitive delay would be clinically valuable, and it might offer guidance for focused interventions in high-risk infants. Currently, such predictions are not feasible at an individual level. Our work corroborates prior literature on epilepsy as a network disease, and the present findings suggest network metrics that may serve as functional biomarkers of neurocognitive development in infants with epilepsy.

## Supplementary Material

fcac295_Supplementary_Data

## Data Availability

All the data needed to evaluate the paper’s conclusions are present in the paper. Additional data may be found in the supplements. Raw connectivity matrices and grouping data needed to reproduce the results in the paper are available on request. MATLAB scripts can be found on the GitHub repository ‘SamiAuno/epi_infant’.

## Abbreviations

AAC =: amplitude-amplitude correlation
dwPLI =: debiased weighted phase lag index
Fc =: central frequency
oCC =: orthogonal correlation coefficient
PPC =: pahse-phase correlation
