# Correction: Networks of cortical activity in infants with epilepsy

**DOI:** 10.1093/braincomms/fcad150

**Published:** 2023-05-11

This is a correction to: Sami Auno, Henna Jonsson, Tarja Linnankivi, Anton Tokariev, Sampsa Vanhatalo, Networks of cortical activity in infants with epilepsy, *Brain Communications*, Volume 4, Issue 6, 2022, fcac295, https://doi.org/10.1093/braincomms/fcac295

In the originally published version of this manuscript, there were errors in the presentation of the figures within the third column and the clarity of that column’s heading within Table 1. This should read:

**Table 1:** Clinical information of the subjects. The number of subjects, the gender ratio, and the mean age at one year EEG in the different subgroups.

Subgroup	Number of subjects	Gender (% female)	Age at 1 year EEG (months ± SD)
All subjects	49	51.0	12.5 ± 0.8
Epilepsy syndrome			
Self-limited	12	41.7	12.4 ± 0.8
West syndrome	27	48.1	12.5 ± 0.6
Unclassified focal	8	62.5	12.3 ± 0.8
Other	2	100.0	12.9 ± 0.8
Neurocognitive development 1 year			
Typical	20	45.0	12.4 ± 0.9
Mild	10	80.0	12.3 ± 0.8
Severe	19	42.1	12.7 ± 0.5
Neurocognitive development 2 years			
Typical	14	50.0	12.4 ± 0.9
Mild	10	50.0	12.4 ± 0.8
Severe	25	52.0	12.6 ± 0.5

instead of:

**Table 1:** Clinical information of the subjects. The number of subjects, the gender ratio, and the mean age at 1 year EEG in the different subgroups

Subgroup	Number of subjects	Gender (% of female)	Age at 1 year EEG (months ± SD)
All subjects	49	51 (0)	12.5 ± 0.8
Epilepsy syndrome			
Self-limited	12	41 (7)	12.4 ± 0.8
West syndrome	27	48 (1)	12.5 ± 0.6
Unclassified focal	8	62 (5)	12.3 ± 0.8
Other	2	100 (0)	12.9 ± 0.8
Neurocognitive development 1 year			
Typical	20	45 (0)	12.4 ± 0.9
Mild	10	80 (0)	12.3 ± 0.8
Severe	19	42 (1)	12.7 ± 0.5
Neurocognitive development 2 years			
Typical	14	50 (0)	12.4 ± 0.9
Mild	10	50 (0)	12.4 ± 0.8
Severe	25	52 (0)	12.6 ± 0.5

This has been corrected in the article.

