# Supplementary material for: Networks of cortical activity in infants with epilepsy
Source: Brain Commun. 2022 Nov 5;4(6):fcac295. doi: 10.1093/braincomms/fcac295 (PMC9692198; doi:10.1093/braincomms/fcac295)
Supplement: fcac295_Supplementary_Data [file fcac295_supplementary_data.pdf]

Networks of cortical activity in infants with epilepsy

Sami Auno[1, 2, 3, 4, †], Henna Jonsson[3, 5], Tarja Linnankivi[3, 5], Anton Tokariev[1, 2, 4, †] and Sampsa Vanhatalo[1, 2, 4, †]

[†]**These authors contributed equally to this work.**

Author affiliations:

1 BABA Center, Department of Clinical Neurophysiology, Children's Hospital, Helsinki University Hospital, Helsinki, 00029 HUS, Finland

2 Neuroscience Center, Helsinki Institute of Life Science, University of Helsinki, 00014 Helsinki, Finland

3 Epilepsia Helsinki, University of Helsinki and Helsinki University Hospital, Helsinki, 00029 HUS, Finland

4 Department of Physiology, University of Helsinki, 00014 Helsinki, Finland

5 Department of Pediatric Neurology and Pediatric Research Center, New Children's Hospital, Helsinki University Hospital and University of Helsinki, Helsinki, 00029 HUS, Finland

Correspondence to: Sami Auno

BABA center, Children's Hospital, Helsinki University Hospital, P.O. Box 281, Stenbäckinkatu 11, 00029 HUS, Helsinki, Finland.

sami.auno@helsinki.fi

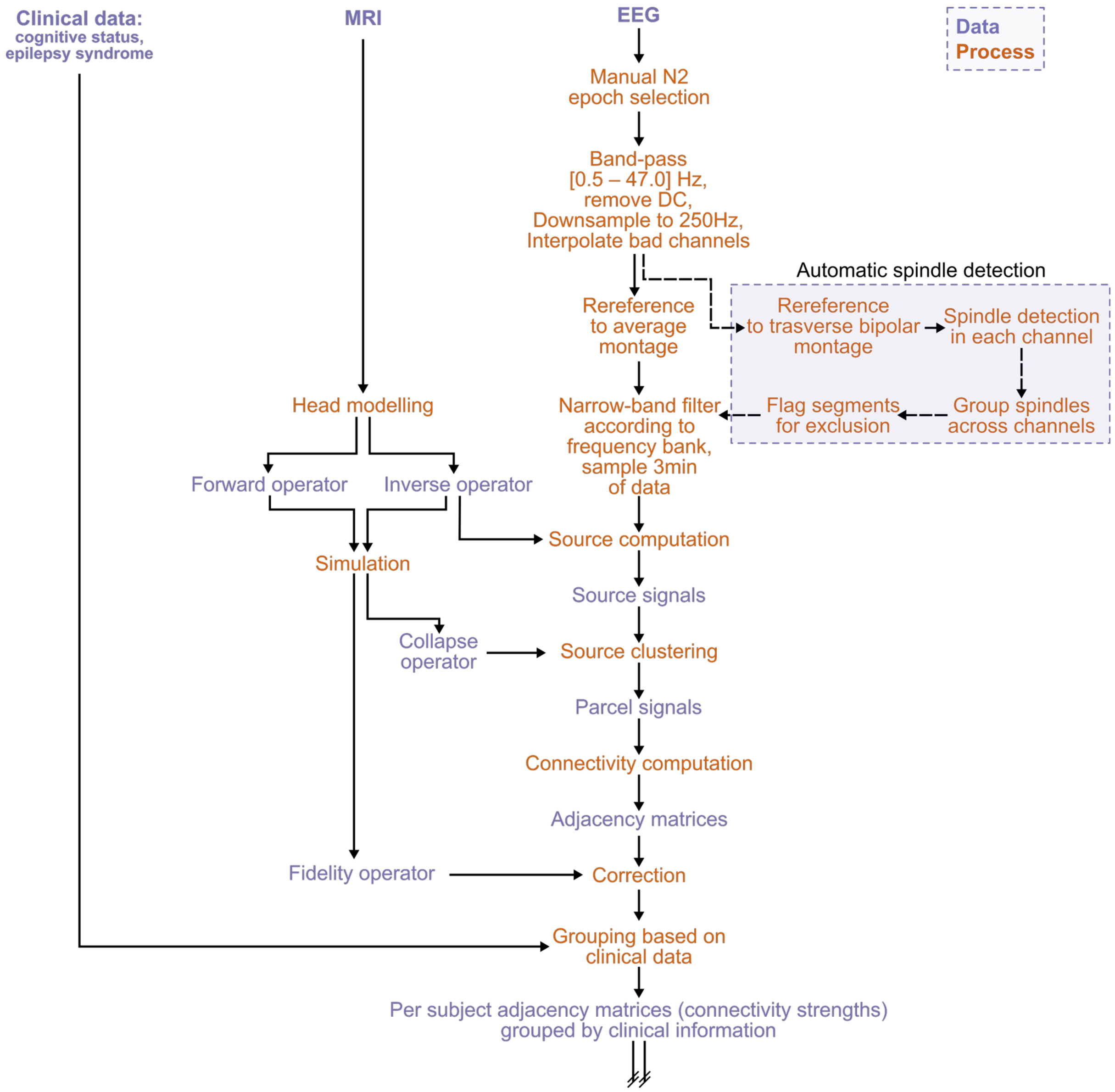

Supplementary Figure 1: Flowchart showing the data processing pipeline up until the analysis pipeline. Clinical and EEG data were recorded in-house. For the MRI based head model we used a common head model based on 90 MRI scans of 1 year-old-infants available online[5].

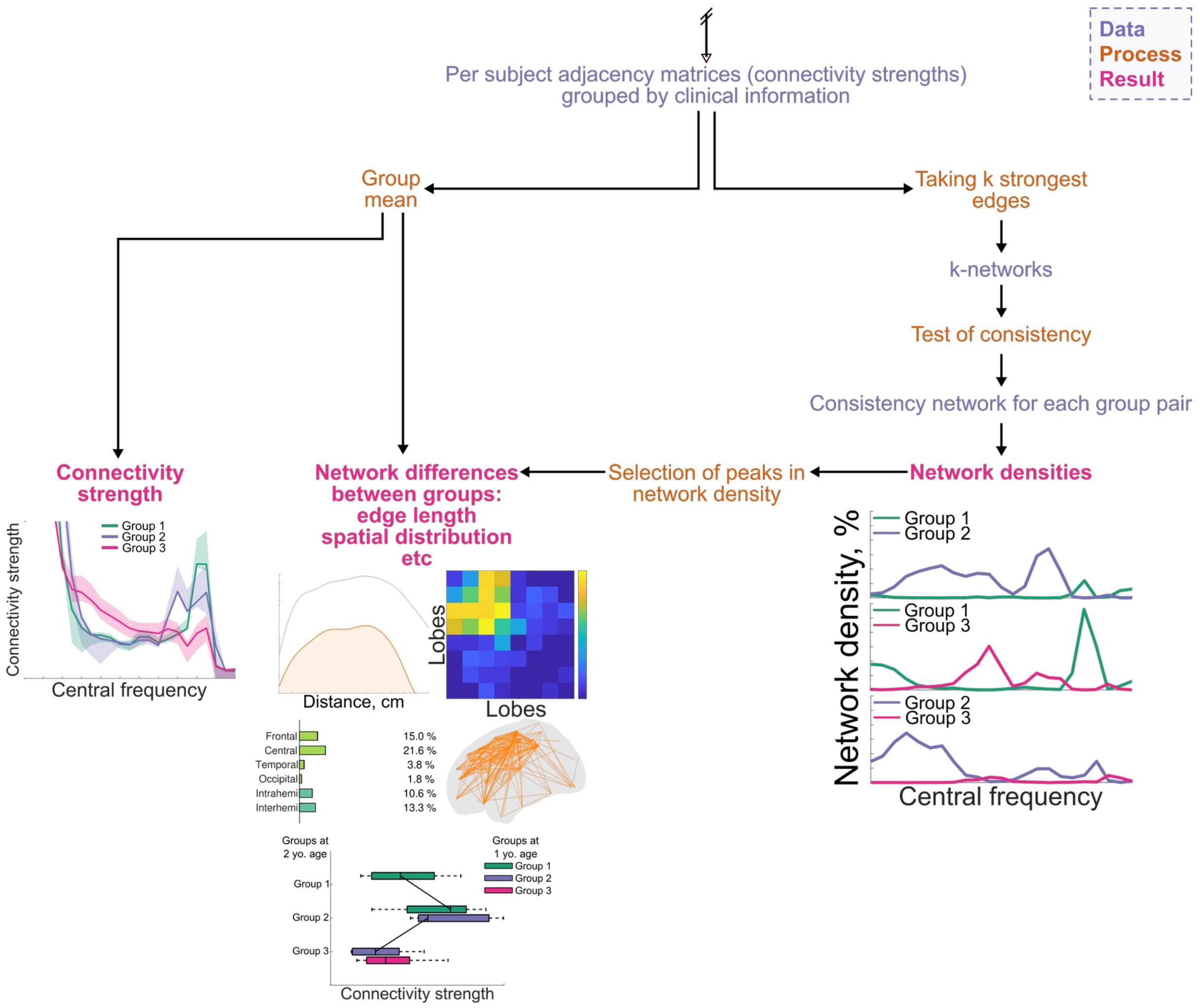

Supplementary Figure 2: Flowchart showing the analysis pipeline after preprocessing.

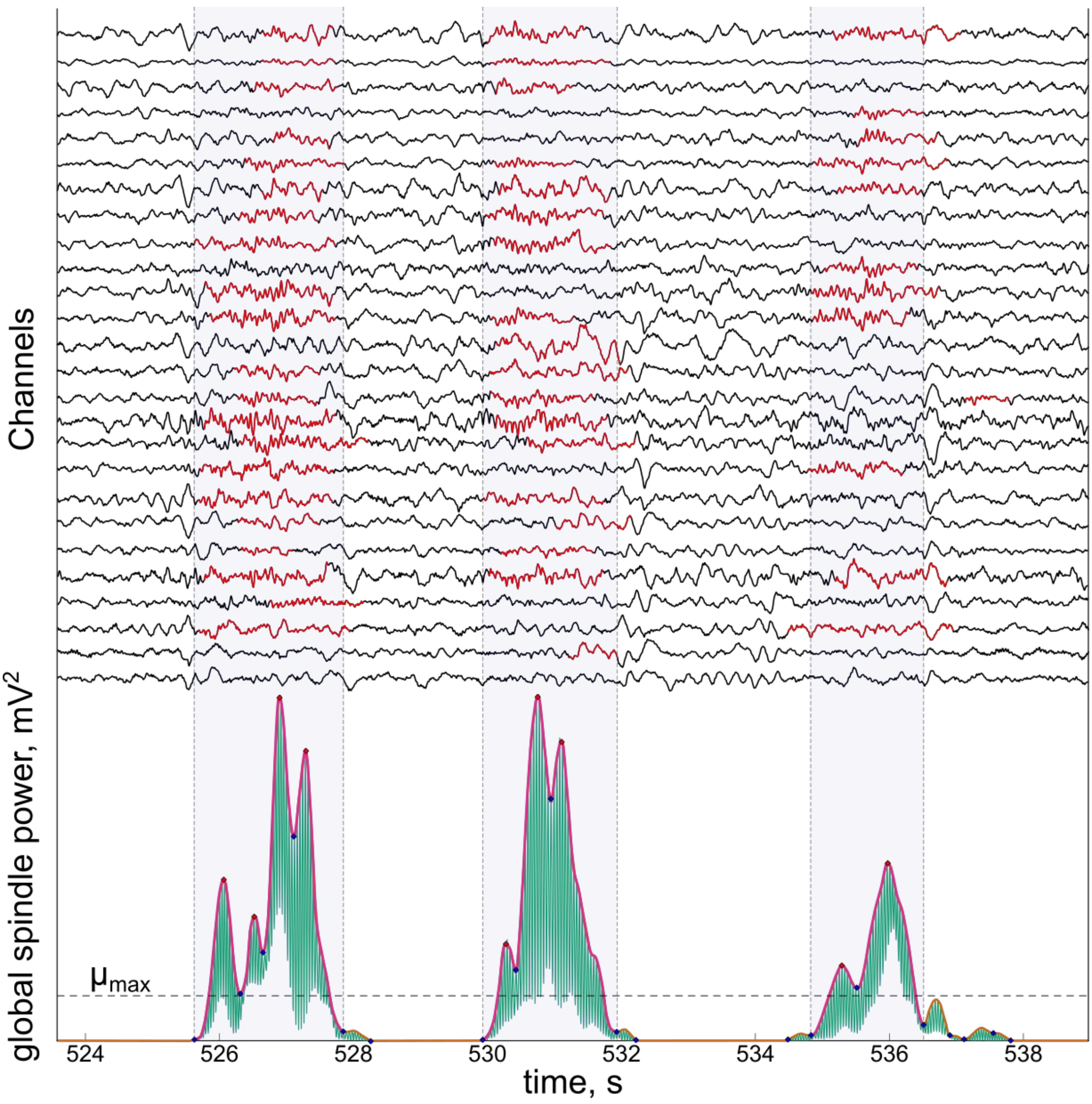

Supplementary Figure 3: Visual representation of sleep spindle exclusion algorithm. The spindles were automatically detected from each channel independentely (red emphasis on channels). The power of the sleep spindles was calculated and summed together (global spindle power, green line). The envelope over the global spindle power was calculated using Akima piecewise interpolation (orange and magenta lines). Each local maxima (red diamond) and local minima (blue diamond) were located. We calculated the mean of all the local maxima (μ_{max}) and rejected those local maxima that were below this mean. Continuous envelopes with alternating local minimas and maximas that started and ended to a local minima (magenta line) were considered and marked as part of a spindle group.

Sleep spindle detection

We applied a modified version of the automated sleep spindle detection algorithm that has been widely used in previous studies[1–4]. To briefly summarise the spindle detection algorithm, the EEG data were first band-pass filtered at 11– 15 Hz with a 2nd order Chebyshev filter (Supplementary Figure 1), and the amplitude envelopes were extracted via Hilbert transform. Then, a channel-wise spindle detection was run using a threshold of three times the standard deviation of the amplitude fluctuation of the mean channel amplitude.[3]

Excluding spindles from the data channel by channel would have introduced discontinueties that probably would have adversely affected the subsequent source reconstruction and analysis. Therefore, we grouped the spindles along the channels and considered only these spindle groups in further analyses and ignored single channel spindle events (Supplementary Figure 3). We performed this spindle clustering by calculating the sum of spindle power over all channels (global spindle power), followed by computing the envelope of the global spindle power by interpolating the values between the local maxima using modified Akima piecewise interpolation. We then determined the mean of the local maxima of the power envelope and rejected all local maxima that were below this mean. A spindle group was defined as a continuous power envelope starting from and ending to a local minimum with alternating local minima and maxima.

Excluding spindles from the epochs was done by excluding whole spindle groups and all data within the spindle group rather than individual spindles. The removal of spindle groups was by simply cutting the spindle group epochs off of the data.

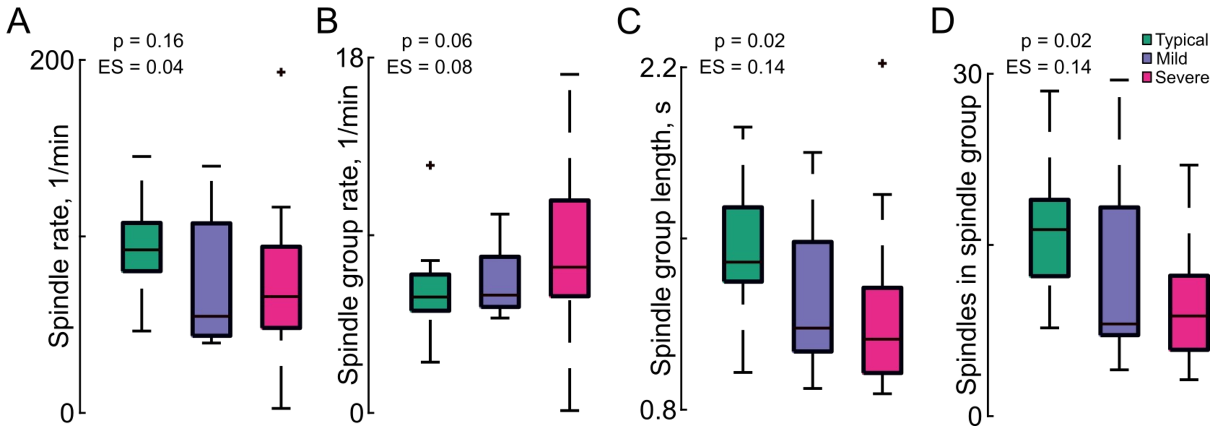

Supplementary Figure 4: Group differences in spindle detection. Subfigure (**A**) shows the number of spindles per minute in each group. The spindles were grouped into spindle groups as explained in Supplementary Figure 3. The number of spindle groups per minute in each group is shown in (**B**). (**C**) shows the spindle group length distribution in each group, whereas the average number of spindles in spindle groups are displayed on subfigure (**D**). The black crosses in the subfigures represent outliers of the whisker plots.

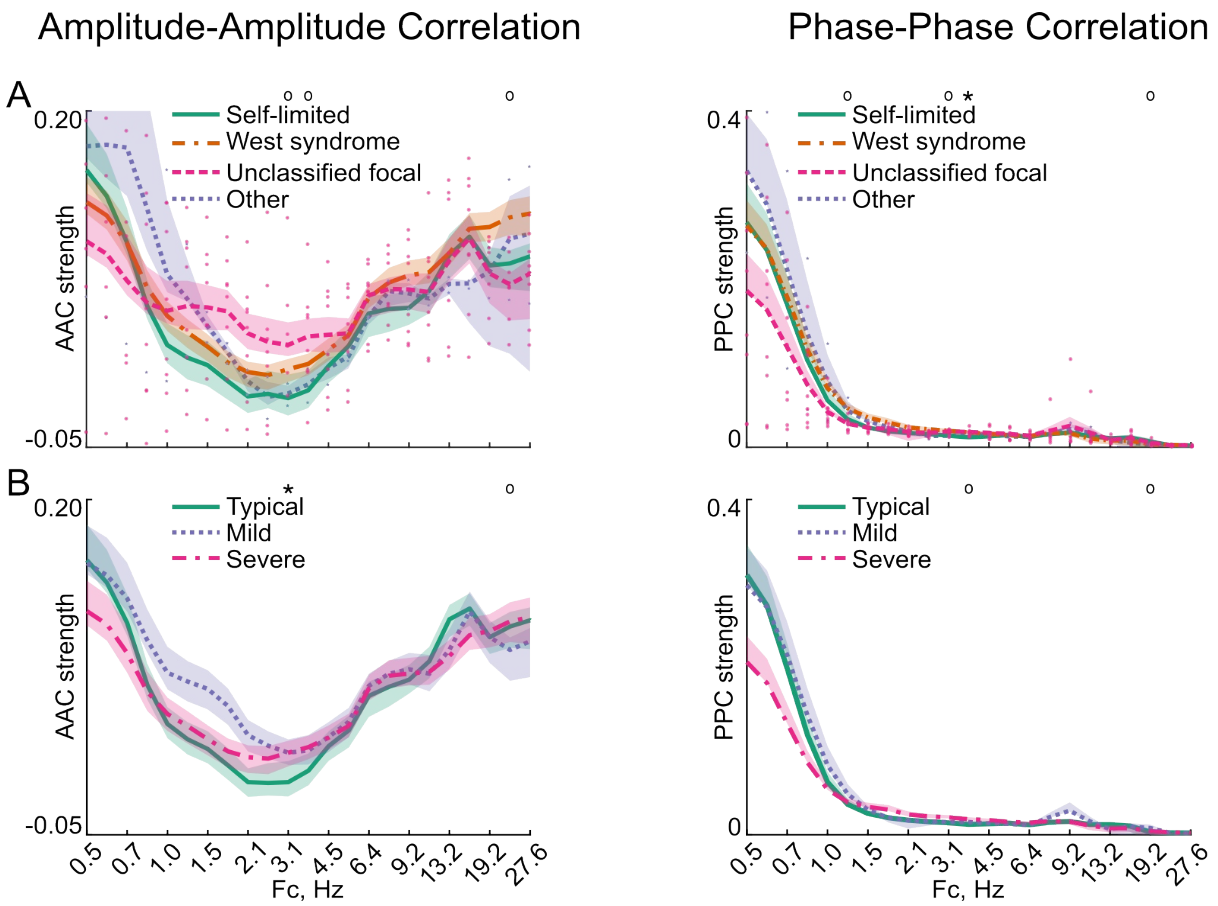

Supplementary Figure 5: Connectivity strength as a function of central frequency band after spindle removal. Global mean amplitude-amplitude (AAC) and phase-phase correlation (PPC) strengths in the four epilepsy syndrome groups (**A**) and the three neurocognitive development groups (**B**) as a function of the central frequency band (*Fc*). For groups with under 10 subjects individual data points are shown. Pink dots indicate subjects in the Unclassified focal group (8 subjects) and the violet stars indicate subjects of the Other group (2 subjects). The neurocognitive groups were determined at the age of two years. The asterix indicates the *Fc* at which the difference between the groups is significant with $P < 0.05$, whereas o indicates $P < 0.1$. *P*-values were determined with Kruskal-Wallis one-way ANOVA.

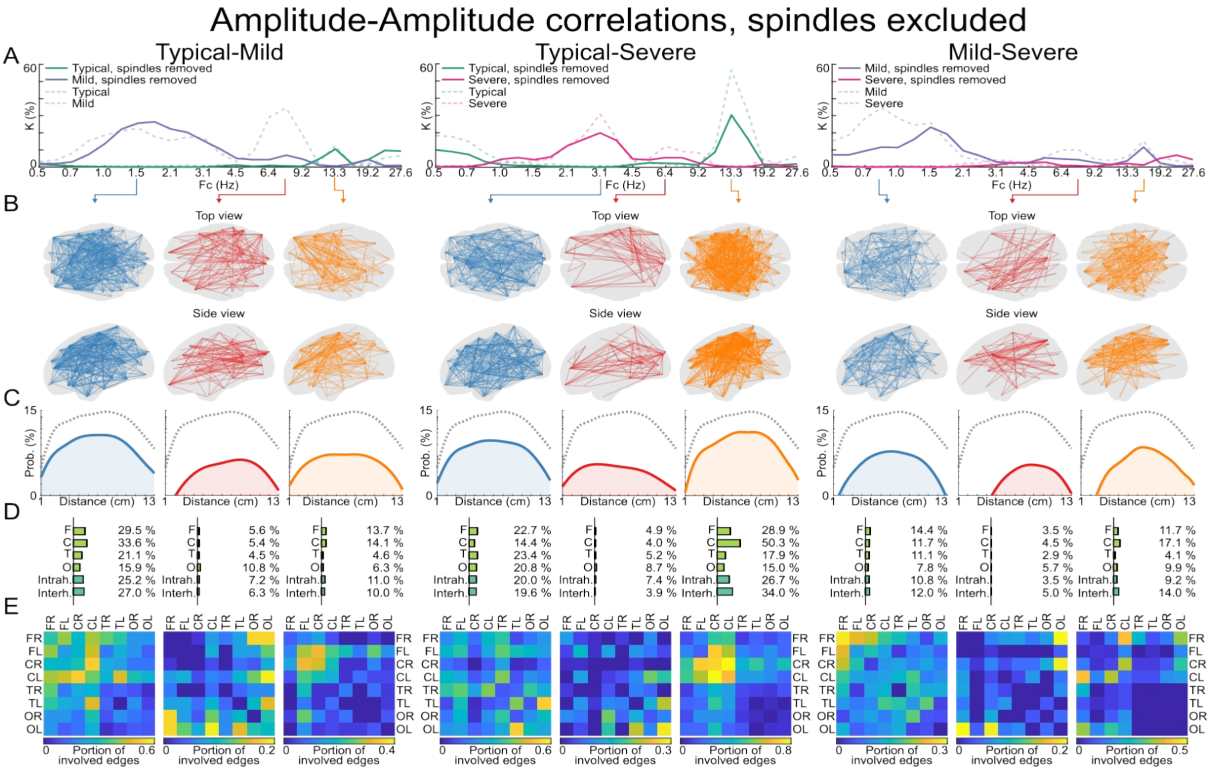

Supplementary Figure 6: Group comparison in amplitude-amplitude correlations (AAC) after spindle removal. (**A**) shows the portion of the edges (density, *K*) that are significantly stronger in the given group when compared to the other group specified. The dashed line shows the density before spindle removal. The statistical analyses were performed with Wilcoxon rank sum test for equal medians with *P*-value threshold of 0.05 and corrected for false discovery rate with Benjamini-Hochberg method. (**B**) shows the significantly stronger edges on a glass-brain. In (**C**), the distance of the edges are shown on logarithmic scale. (**D**) shows the portion of the nodes that are involved in the given network. 100 % involvement would signify that all possible edges from the nodes of the given lobe are part of the significant network. Likewise in (**E**), the portion of the edges between two given regions that are part of the significant network. 1.0 signifies that all edges are part of the significant network. Abbreviations: C = central lobe; F = frontal lobe; Fc = central frequency; L = left; O = occipital lobe; R = right; T = temporal lobe; Inter h. = inter hemispheric; Intra h. = intra hemispheric.

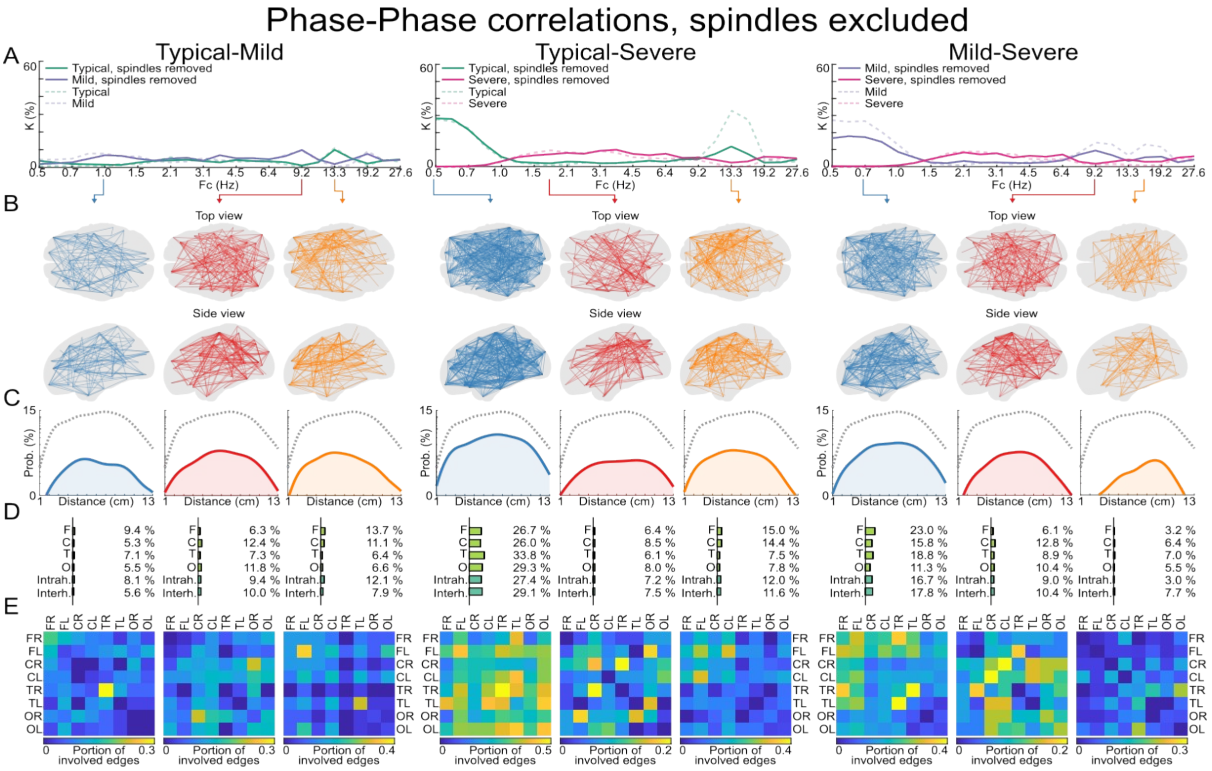

Supplementary Figure 7: Group comparison in phase-phase correlations (PPC) after spindle removal. (**A**) shows the portion of the edges (density, *K*) that are significantly stronger in the given group when compared to the other group specified. The dashed line shows the density when spindles have been removed from the data prior to analysis. The statistical analyses were performed with Wilcoxon rank sum test for equal medians with *P*-value threshold of 0.05 and corrected for false discovery rate with Benjamini-Hochberg method. (**B**) shows the significantly stronger edges on a glass-brain. In (**C**), the distance of the edges are shown on logarithmic scale. (**D**) shows the portion of the nodes that are involved in the given network. 100 % involvement would signify that all possible edges from the nodes of the given lobe are part of the significant network. Likewise in (**E**), the portion of the edges between two given regions that are part of the significant network. 1.0 signifies that all edges are part of the significant network. Abbreviations: C = central lobe; F = frontal lobe; Fc = central frequency; L = left; O = occipital lobe; R = right; T = temporal lobe; Inter h. = inter hemispheric; Intra h. = intra hemispheric.

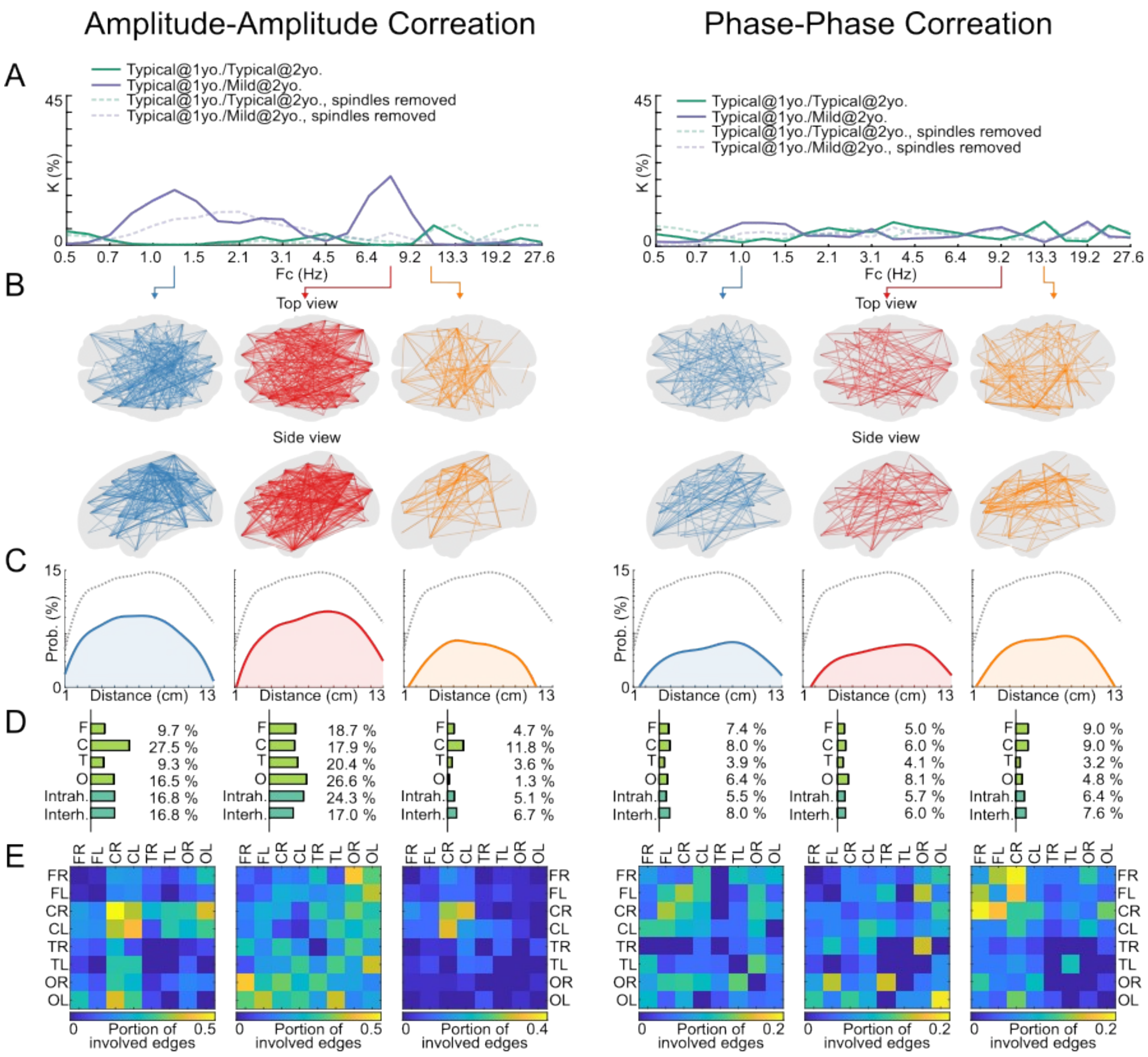

Supplementary Figure 8: Group comparison with in amplitude-amplitude correlations (AAC) with change-in-cognitive-development groups. (**A**) shows the portion of the edges (density, *K*) that are significantly stronger in the given group when compared to the other group specified. The dashed line shows the density before spindle removal. The statistical analyses were performed with Wilcoxon rank sum test for equal medians with *P*-value threshold of 0.05 and corrected for false discovery rate with Benjamini-Hochberg method. (**B**) shows the significantly stronger edges on a glass-brain. In (**C**), the distance of the edges are shown on logarithmic scale. (**D**) shows the portion of the nodes that are involved in the given network. 100 % involvement would signify that all possible edges from the nodes of the given lobe are part of the significant network. Likewise in (**E**), the portion of the edges between two given regions that are part of the significant network. 1.0 signifies that all edges are part of the significant network. Abbreviations: C = central lobe; F = frontal lobe; Fc = central frequency; L = left; O = occipital lobe; R = right; T = temporal lobe; Inter h. = inter hemispheric; Intra h. = intra hemispheric.

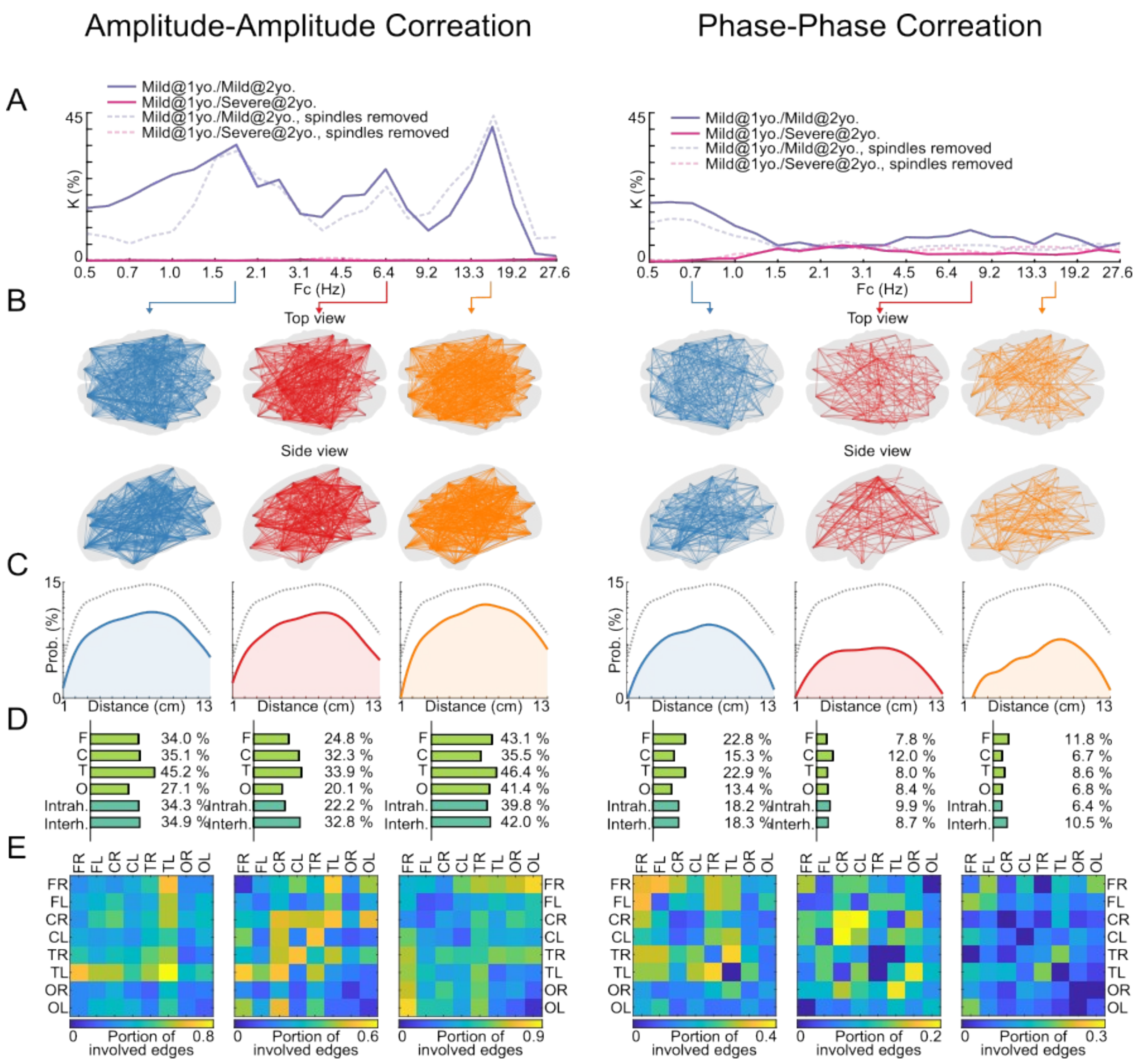

Supplementary Figure 9: Group comparison with in phase-phase correlations (PPC) with change-in-cognitive-development groups. (**A**) shows the portion of the edges (density, *K*) that are significantly stronger in the given group when compared to the other group specified. The dashed line shows the density before spindle removal. The statistical analyses were performed with Wilcoxon rank sum test for equal medians with *P*-value threshold of 0.05 and corrected for false discovery rate with Benjamini-Hochberg method. (**B**) shows the significantly stronger edges on a glass-brain. In (**C**), the distance of the edges are shown on logarithmic scale. (**D**) shows the portion of the nodes that are involved in the given network. 100 % involvement would signify that all possible edges from the nodes of the given lobe are part of the significant network. Likewise in (**E**), the portion of the edges between two given regions that are part of the significant network. 1.0 signifies that all edges are part of the significant network. Abbreviations: C = central lobe; F = frontal lobe; Fc = central frequency; L = left; O = occipital lobe; R = right; T = temporal lobe; Inter h. = inter hemispheric; Intra h. = intra hemispheric.

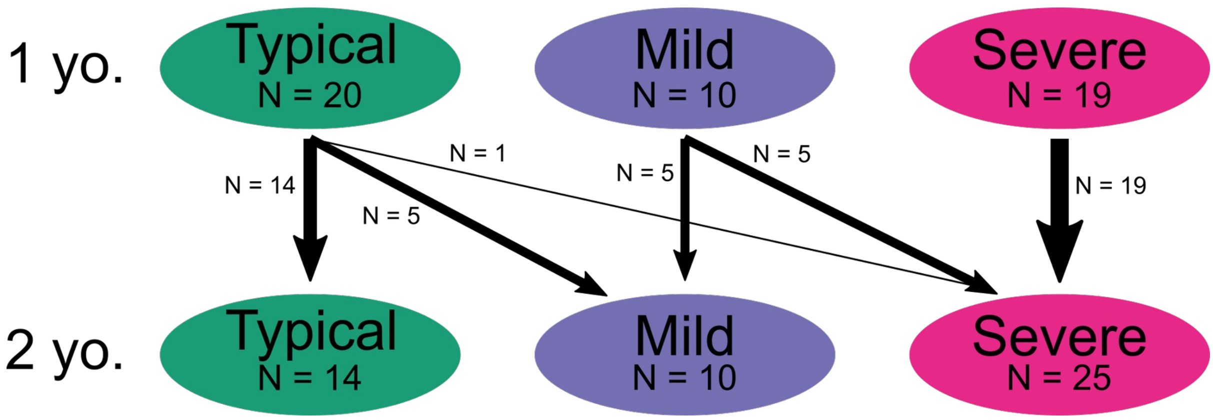

Supplementary Figure 10: Number of subjects in each neurocognitive development groups and the associated changes between 1 and 2 years of age.

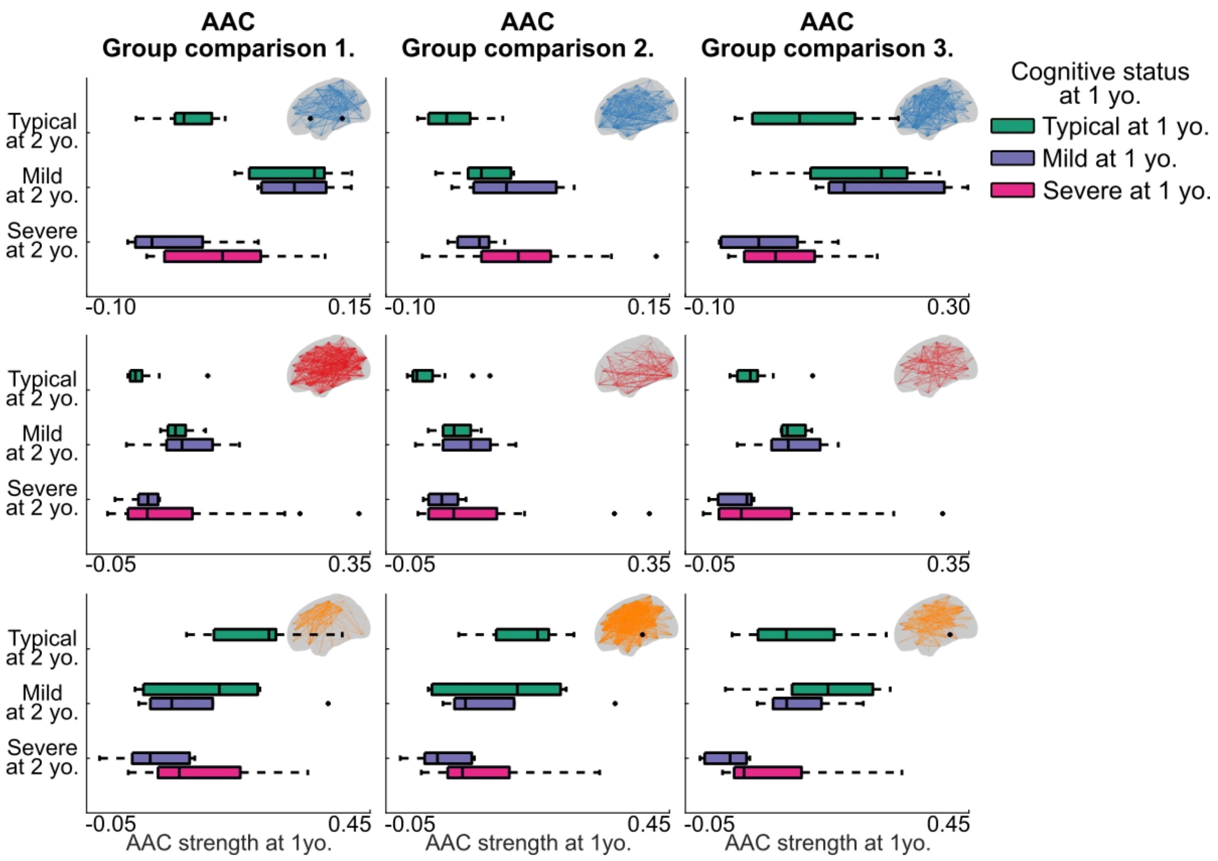

Supplementary Figure 11: Comparing the AAC strength between groups by change in cognitive status. The subjects have been grouped into five groups based on their cognitive status at 1 year and 2 years-of-age. The colors of the bars signify the age at 1 year-of-age. The cognitive status at 2 years-of-age is labelled on the y-axis. The connectivity strength is the mean over the shown edges (glass brain on upper right corner). The edges and related central frequencies are presented in Supplementary Figure 6.

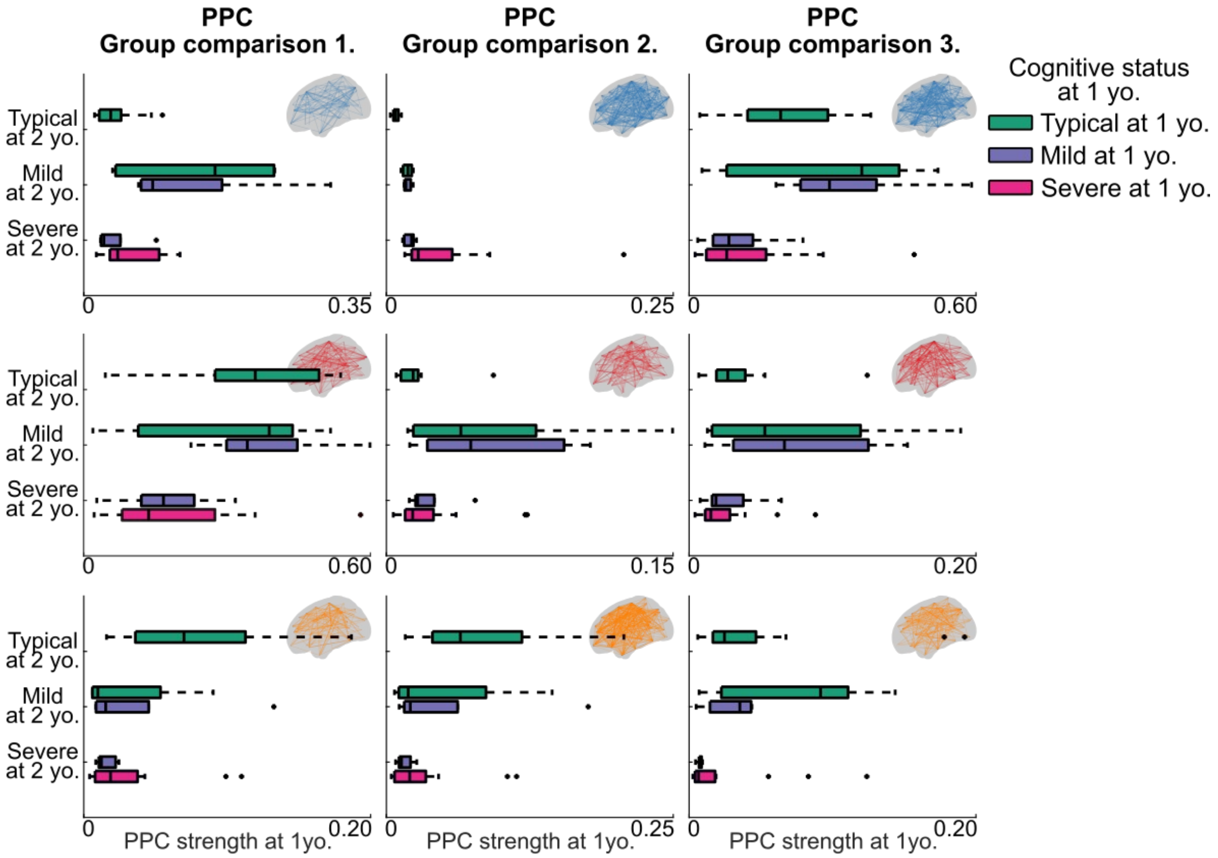

Supplementary Figure 12: Comparing the PPC strength between groups by change in cognitive status. The subjects have been grouped into five groups based on their cognitive status at 1 year and 2 years-of-age. The colors of the bars signify the age at 1 year-of-age. The cognitive status at 2 years-of-age is labelled on the y-axis. The connectivity strength is the mean over the shown edges (glass brain on upper right corner). The edges and related central frequencies are presented in Supplementary Figure 7.

Supplementary Table 1 Subject syndromes and cognitive development at 1 and 2 years of age

Subject number	Gender	Age at 1 year EEG (months)	Syndrome	Cogn. dev. 1yo.	Cogn. dev. 2yo.
1	F	12,0	Unclassified focal	Mildly impaired	Severily impaired
2	M	12,0	West syndrome	Severily impaired	Severily impaired
3	F	12,5	Self-limited	Typical	Typical
4	M	12,1	West syndrome	Severily impaired	Severily impaired
5	F	12,2	West syndrome	Severily impaired	Severily impaired
6	M	12,6	Self-limited	Typical	Typical
7	M	12,1	West syndrome	Typical	Mildly impaired
8	F	13,4	Unclassified focal	Typical	Mildly impaired
9	M	12,5	Self-limited	Typical	Typical
10	F	12,6	Other	Mildly impaired	Severily impaired
11	F	13,1	West syndrome	Severily impaired	Severily impaired
12	F	12,0	Self-limited	Typical	Typical
13	M	12,2	West syndrome	Severily impaired	Severily impaired
14	F	12,1	Unclassified focal	Mildly impaired	Mildly impaired
15	F	12,7	West syndrome	Severily impaired	Severily impaired
16	M	12,2	West syndrome	Severily impaired	Severily impaired
17	M	12,3	Self-limited	Typical	Typical
18	M	15,5	West syndrome	Severily impaired	Severily impaired
19	F	12,2	West syndrome	Typical	Mildly impaired
20	M	12,4	Self-limited	Typical	Mildly impaired
21	F	11,6	West syndrome	Mildly impaired	Severily impaired
22	F	11,7	Unclassified focal	Typical	Typical
23	M	12,2	West syndrome	Severily impaired	Severily impaired
24	F	12,7	Self-limited	Typical	Typical
25	F	12,1	West syndrome	Severily impaired	Severily impaired

26	M	12,0	Self-limited	Typical	Typical
27	M	13,6	Unclassified focal	Mildly impaired	Mildly impaired
28	F	12,5	West syndrome	Severily impaired	Severily impaired
29	F	11,8	West syndrome	Typical	Typical
30	F	12,8	Self-limited	Typical	Typical
31	M	12,3	West syndrome	Severily impaired	Severily impaired
32	M	13,8	West syndrome	Severily impaired	Severily impaired
33	F	12,1	West syndrome	Mildly impaired	Mildly impaired
34	F	12,1	Self-limited	Typical	Typical
35	M	12,1	Self-limited	Typical	Mildly impaired
36	M	12,7	West syndrome	Typical	Typical
37	M	11,5	West syndrome	Mildly impaired	Mildly impaired
38	M	13,7	West syndrome	Severily impaired	Severily impaired
39	M	11,7	West syndrome	Severily impaired	Severily impaired
40	F	12,1	Self-limited	Severily impaired	Severily impaired
41	F	12,2	West syndrome	Mildly impaired	Severily impaired
42	M	11,4	Unclassified focal	Severily impaired	Severily impaired
43	M	12,7	West syndrome	Typical	Typical
44	*F*	13,1	Other	Mildly impaired	Severily impaired
45	M	13,4	Self-limited	Typical	Typical
46	F	12,3	West syndrome	Severily impaired	Severily impaired
47	M	12,1	Unclassified focal	Typical	Severily impaired
48	F	12,4	Unclassified focal	Mildly impaired	Mildly impaired
49	F	14,5	West syndrome	Severily impaired	Severily impaired

References

1. Ferrarelli F, Huber R, Peterson MJ, et al. Reduced Sleep Spindle Activity in Schizophrenia Patients. *American Journal of Psychiatry*. 2007;164(3):483-492. doi:10.1176/ajp.2007.164.3.483.

2. Plante DT, Goldstein MR, Landsness EC, et al. Topographic and sex-related differences in sleep spindles in major depressive disorder: A high-density EEG investigation. *Journal of Affective Disorders*. 2013;146(1):120-125. doi:10.1016/j.jad.2012.06.016

3. Sarasso S, Proserpio P, Pigorini A, et al. Hippocampal sleep spindles preceding neocortical sleep onset in humans. *NeuroImage*. 2014;86:425-432. doi:10.1016/j.neuroimage.2013.10.031

4. D'Atri A, Novelli L, Ferrara M, Bruni O, De Gennaro L. Different maturational changes of fast and slow sleep spindles in the first four years of life. *Sleep Medicine*. 2018;42:73-82. doi:10.1016/j.sleep.2017.11.1138

5. Shi F, Yap PT, Wu G, et al. Infant Brain Atlases from Neonates to 1- and 2-Year-Olds. *PLOS ONE*. Published online April 2011. doi:10.1371/journal.pone.0018746
